# Metals Are Integral to Life as We Know It

**DOI:** 10.3389/fcell.2022.864830

**Published:** 2022-03-04

**Authors:** Daniele Rossetto, Sheref S. Mansy

**Affiliations:** ^1^ D-CIBIO, University of Trento, Povo, Italy; ^2^ Department of Chemistry, University of Alberta, Edmonton, AB, Canada

**Keywords:** origins of life, prebiotic chemistry, metal ions, bioinorganic chemistry, metallopeptides

## Abstract

Investigations of biology and the origins of life regularly focus on the components of the central dogma and thus the elements that compose nucleic acids and peptides. Less attention is given to the inorganic components of a biological cell, which are required for biological polymers to function. The Earth was and continues to be rich in metals, and so investigations of the emergence and evolution of life must account for the role that metal ions play. Evolution is shaped by what is present, and not all elements of the periodic table are equally accessible. The presence of metals, the solubility of their ions, and their intrinsic reactivity all impacted the composition of the cells that emerged. Geological and bioinformatic analyses clearly show that the suite of accessible metal ions changed over the history of the Earth; however, such analyses tend to be interpreted in comparison to average oceanic conditions, which do not represent well the many niche environments present on the Earth. While there is still debate concerning the sequence of events that led to extant biology, what is clear is that life as we know it requires metals, and that past and current metal-dependent events remain, at least partially, imprinted in the chemistry of the cell.

The elements necessary for extant biology are frequently referred to as CHNOPS for carbon, hydrogen, nitrogen, oxygen, phosphorous, and sulfur. While undoubtedly necessary for the synthesis of biological molecules, such as nucleic acids, proteins, and lipids, CHNOPS alone is incapable of supporting life as we know it (Figure 1). Nothing alive today or in the past on this planet can be adequately understood without invoking metals. In extant biology, metal ions aid the folding of biological polymers, catalyze the reactions of metabolism, form gradients across membranes that serve as energy reserves, and mediate signal transduction events. Every class of molecules in biology, from nucleic acids to proteins and antibiotics are impacted by metal ions, which is why living cells put so much effort in regulating the concentrations of intracellular free metal ions (Capdevila et al., 2017). What perhaps goes less frequently noticed is the impact of the environment on the distribution and exploitation of metal ions in biology. We are accustomed to viewing biology in the light of evolution and how environmental conditions shape emergent phenotypes. The role of metal ions in biology is no different. Our planet has always been rich in metals, so Darwinian evolution must be thought of in the context of these conditions. Metal ions affect ionic polymers, such as nucleic acids and the soluble domains of proteins, by neutralizing charges and thus facilitating the formation of the tertiary folds necessary for activity. Further, metals themselves intrinsically possess catalytic activity. Since it is easier to scavenge existing parts (i.e. metal ions) for needed function rather than to build from scratch, it is not surprising that metal ions are frequently found within the active sites of enzymes. We see this in both biology and *in vitro* evolution experiments (Bartel and Szostak, 1993; Monreal Santiago et al., 2020), where selected polymers frequently rely on the activity of a coordinated metal ion, even if metal-dependent function was not intentionally sought (Seelig and Szostak, 2007). We know that in many instances metal-independent activity is possible, because the natural metal-dependent activity of some enzymes can be used as a starting point to engineer metal-independent activity (Casareno et al., 1995), and when environments change, metal reliant pathways can evolve to exploit the use of organic cofactors in place of metal ions (Daniel and Danson, 1995). However, metal-dependent catalysis does not require the formation of a stable complex. Instead, metal ions can enter and exit an active site, as needed, to mediate turnover. Such a scenario is observed for *Bacillus halodurans* RNase H, which exploits for catalysis a transient Mg^2+^ and two transient K^+^ that do not make direct contact with the protein (Samara and Yang, 2018). Such dynamics are possible because the intracellular concentrations of Mg^2+^ and K^+^ are high, so high affinity, static binding is not necessary.

**FIGURE 1:**
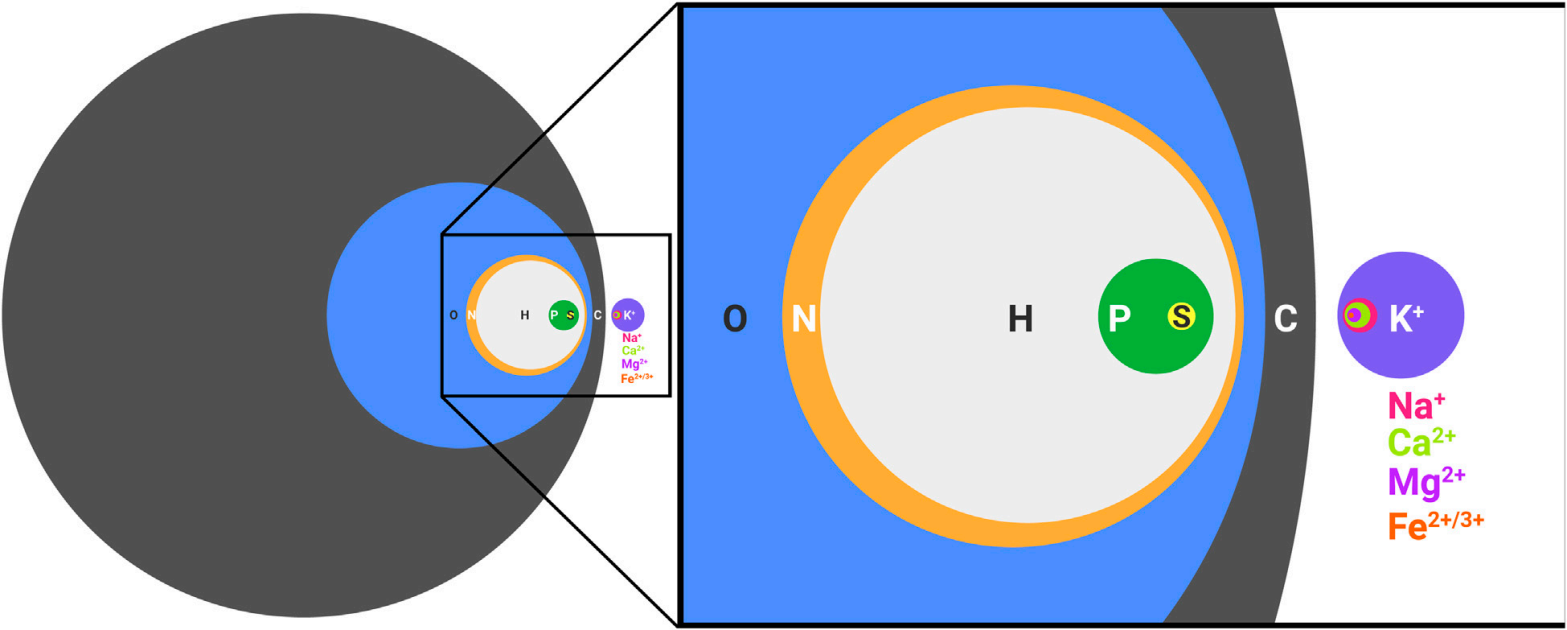
CHNOPS and the most abundant metal ions of a biological cell. Diameters are proportional to percent dry weight. Data are of an average bacterial composition and taken from Lawford and Rousseau, 1996; Tchobanoglous Burton, Franklin L., Stensel, H. David, Metcalf and Eddy, 2003.

If biology exploits what is accessible, then it is reasonable to ask which metal ions were present. Relative abundancies throughout the universe roughly correlate with atomic number, with smaller elements of even atomic number found in greater abundance than larger elements of odd atomic number (Da Silva and Williams, 2001). However, planetary compositions, unsurprisingly, are different from each other due to the impact of gravity and heat from the sun, leading to smaller, metal-rich planets closer to the sun, such as the Earth, and larger, more gaseous planets with lower metal abundancies further away. Further deviations result from meteoritic impacts. The metals of greatest abundance on the Earth are the alkali and alkaline Earth metals of periods two to four and the first-row transition metals. Trivalent and higher valent metal cations are not typically encountered, because of their precipitation as hydroxides and oxides. Therefore, solubility limits the accessible options to mono- and di-valent cations. A clear example of this is the change in concentration of oceanic iron before (∼10^–7^ mM Fe^2+^) and after (∼10^–19^ mM Fe^3+^) the great oxidation event. The appearance of oxygen also altered the ratios of sulfate to sulfide, which in turn affected the solubility of metal ions. Metal sulfates are highly soluble in comparison to metal sulfides, and so the concentrations of some metal ions, such those of copper and zinc, increased after the Earth became aerobic. In fact, copper and zinc-dependent enzymes are found much more frequently in higher organisms that emerged after the great oxidation event. While loss of a specific metal ion could be detrimental, that was likely not always the case. Some enzymes retain function with different metal ions. For example, some forms of superoxide dismutase (SOD) are functional as Fe^2+^- or Mn^2+^-bound protein (Meier et al., 1982). Similar promiscuity was likely common in the past, particularly with Fe^2+^, which could have been used in place of Mg^2+^ (Moore et al., 2017; Okafor et al., 2017). The common binding motifs between iron-sulfur clusters and Zn^2+^ also suggest that more ancient scaffolds may have been used for the coordination of newly available metal ions (Belmonte and Mansy, 2017; Shimberg et al., 2018). Although oceanic concentrations of metal ions fluctuated over the history of the Earth, some metal ions, such as Na^+^, K^+^, Mg^2+^, and Ca^2+^, are soluble as sulfides and oxides, and thus may not have changed much.

While it is instructive to assess the compositional evolution of the ocean, oceanic conditions are not where all life evolved. The planet is highly varied in composition and conditions, and so care must be taken when inferring past events based on average conditions of the ocean. For example, despite the much lower abundance of molybdenum (∼100-fold lower concentration) in the sea of the anaerobic Earth in comparison to iron, evolutionary analysis indicates that Mo-dependent nitrogenase predates Fe-dependent nitrogenase (Kacar et al., 2021). This suggests that such enzymes emerged from organisms in niche environments not well represented by average oceanic conditions. The same is likely true for the emergence of the Earth’s first cells, which likely did not occur under the average conditions of the Earth. Several competing theories exist, with prebiotic chemistry of surface lake conditions being the most intensely investigated (Sasselov et al., 2020). Lake conditions are attractive since lakes can harness the energy of the sun and can keep molecules necessary for life, such as phosphate, soluble. Lakes rich in carbonate would have led to the precipitation of complexes with Mg^2+^ and Ca^2+^, thus decreasing the concentrations of these metal ions, which would have facilitated the formation of protocellular structures (Toparlak et al., 2020) and allowed for phosphate concentrations higher than one molal (Toner and Catling, 2020). While decreasing the concentration of Mg^2+^ may have been helpful for some chemical steps, Mg^2+^ would have facilitated other necessary reactions. Mg^2+^-binding sites are postulated to be older than the last universal common ancestor (LUCA), and at least 18% of extant gene products are thought to bind Mg^2+^ (Shalaeva et al., 2018).

Since the enzymes that mediate metabolism are heavily reliant on metal cofactors, many suspect that prebiotic analogues of extant metabolism may have operated on the prebiotic Earth in a metal-dependent fashion. The degree of involvement of metal ions is debated, with some advocating for a strong role in catalyzing glycolysis (Keller et al., 2014) and the citric acid cycle (Muchowska et al., 2019), and others advocating for a diminished role (Stubbs et al., 2020). Nevertheless, metal ions must have impacted prebiotic chemistry, as it is difficult to imagine environments completely devoid of metals. Copper (Patel et al., 2015) and iron (Xu et al., 2018) ions have been invoked in cyanosulfidic protometabolic pathways that synthesize RNA, amino acids, and lipid precursors. The prebiotic synthesis of phosphoenol pyruvate exploits manganese (Coggins and Powner, 2017), and the non-enzymatic copying and ligation of RNA strands relies on Mg^2+^ (Adamala and Szostak, 2013). More complex metallocofactors, such as iron-sulfur clusters, can be synthesized prebiotically (Bonfio et al., 2017), retain redox activity when bound to small peptides (Scintilla et al., 2016; Kim et al., 2018), and can engage in electron transfer reactions that generate a proton gradient across a lipid membrane (Bonfio et al., 2018).

Billions of years of evolution have given rise to organisms that are supported by finely tuned chemistry, which can be seen by how metals are used in biology. Signal transduction makes use of metal ions with fast ligand exchange rates, such as Na^+^, K^+^, and Ca^2+^ (Cowan, 1997). Enzymes exploit the Lewis acidity of metal ions, e.g. Zn^2+^, to mediate reactions with small substrates that would be difficult to achieve with only protein sidechains (Da Silva and Williams, 2001). However, the origins of the observed metal dependencies reflect what is or was readily accessible. An instructive example of how metal dependence can become embedded is seen by the lengths that modern organisms, including pathogenic bacteria (Capdevila et al., 2017), go to acquire Fe^2+^. Ultimately, life relies on a conserved set of metal ions in a way that is not too dissimilar from the well-recognized dependencies on a shared genetic code and a common central metabolism. What is less clear is the extent that each of these central pillars of biology rely on each other, and similarly, if life must be constructed from these same parts. It is easy to propose that different planetary conditions could give rise to life completely orthogonal to ours today, but it is more difficult to imagine when considering the limits of availability and accessible chemistry (Pace, 2001). To date, few have attempted to define the metal requirements for the emergence of life (Zerkle et al., 2005; McKay, 2014; Barge et al., 2021). Although evolution suggests that there is unlikely to be a requirement for one specific metal, the probability of prebiotic chemistry advancing towards cell-like activity may be significantly lower in the absence of the intrinsic catalytic activity of metal ions in general. As the search for extraterrestrial life focuses on rocky planets, there is already a presumption to the importance of metals. The variable, instead, is accessibility.
